# Necrology

**Published:** 1901-11

**Authors:** 


### NECROLOGY.

T. F. Mullaney, of Kansas City, Mo., died on September 27, 1901, after a brief illness, from appendicitis. Surgical intervention failed to give relief.

Dr. Mullaney was not a graduate of any college, though he had served an apprenticeship period and was an eager seeker after knowledge, following always the progress of veterinary science as presented in the journalism of our country. He was City Veterinarian of Kansas City, and had secured for this position a generous recognition by his city and a fitting compensation for the place. Those who met him at Atlantic City will recall the hustling character he presented. Though successful to a marked degree in practice, he had planned to secure a college degree and proposed entering one of the schools of our country. He leaves a wife and several small children.

Dr. Frank T. Shannon, graduate of the Veterinary Department of the University of Pennsylvania, class of 1894, an attaché of the Bureau of Animal Industry, died at Pueblo, Colo., August 9, 1901, of typhoid fever. Dr. Shannon had been connected with the Bureau of Animal Industry almost all the period from the date of his graduation, and had been connected with almost every branch of the service, and travelled over almost our entire country in this work.

ONTARIO VETERINARY COLLEGE, TORONTO, CANADA. The opening lecture was delivered in the lecture-room of the college by Prof. Andrew Smith, the principal, at 2 P.M., on Wednesday, October 16th. A large number of students, consisting of undergraduates and freshmen, were present—an encouraging evidence of the returning interest in veterinary science and an expression of appreciation of this widely known school of veterinary medicine.

